# Quarterly Summary of the Transactions of the College of Physicians for August, September and October, 1842

**Published:** 1842-11-12

**Authors:** 


*Quarterly Summary of the Transactions of the College of Physicians for August, September and October*, 1842.

This pamphlet is taken up mainly with the "recapitulation and conclusion" of a lengthy paper on *Mesmerism*, read before the College of Physicians, by Dr. J. K. Mitchell. This recapitulation comprises the deductions of the author from a series of facts collected from a personal examination of the subject during the last five years. The high character of Dr. Mitchell, and the obvious spirit of fairness which characterizes his investigation, give an interest to what he says on this topic which induces us to copy at length his "recapitulation." It will be seen that Dr. M. rejects the delusions of *phreno-mesmerism* and *clairvoyance*, as totally destitute of foundation in fact, but that he believes that the phenomena of the mesmeric state "cannot be accounted for by imagination and imitation," and are "not to be ascribed to any thing but a physical influence." This mesmeric influence he attributes to a nervous *induction*, analogous to the action of *presence* in chemistry. As we learn that Dr. Mitchell is about to publish a work on the subject, we are indisposed just now to enter upon a discussion of it. In giving place to the following summary of his views, at the present time, we of course are not to be understood to subscribe to the positions assumed. On the contrary, we dissent from every other explanation of the phenomena of mesmerism, than by the workings of the patient's imagination.

*Recapitulation.*

1. The investigations into the claims of mesmerism have been hitherto imperfect, because they have been conducted either by interested partisans, or prejudiced opponents.

2. All previous examinations of this difficult subject have been directed rather to its undue pretensions, than to its less obtrusive foundations.

3. The researches of the committees detailed by learned societies, have been contradictory and unfruitful, chiefly, because the trained subjects of the mesmerizers were examined, instead of those among their own friends and acquaintances, on whom they could rely for the unsophisticated representation of the natural phenomena of mesmerism. They invited deception, and either implicitly confided in it; or, having detected the attempt to mislead, condemned the whole system as one of fraud and imposture. Hence, they were always in those extremes which border on truth, but are never within its confines. Astronomy is not the less true, because the ignorant believe that the stars are holes through which the light of Heaven breaks, or because astrologers pretend to see the fates of humanity registered in the conjunction and disseverance of the planets.

4. Imagination and imitation cannot account for the uniformity of the phenomena of the mesmeric state, in persons of all ages and conditions, who are totally ignorant, not only of the symptoms to be produced, but of the design of the mesmerizer.

5. Neither will they explain the analogies found to exist between natural and artificial somnambulism.

6. Nor can we, by any rational view of their cases, ascribe to any thing but a physical influence, the effect of *passes* on the diseased condition of certain patients, some of whom did not observe the manipulation, and none of whom understood its import.

7. Admitting that the mesmeric sleep may be and is produced solely by mental means, the method as well as the phenomena of restoration, both in natural and artificial somnambulism, forbid us to believe that the patients are usually conscious either of the act or the intention. Many of them showed plainly their ignorance by their conversation at the time, and others were totally incapacitated for observation.

8. If we admit the awakening without the aid of the patient's mental co-operation, we can find no reasonable difficulty in believing that the mesmeric sleep is producible also without that co-operation.

9. The phenomena of artificial somnambulism are, 1st. An exaltation of the circulation, without a corresponding increase of the respiration. 2d. An obtunded sensibility to causes of pain, and sometimes, though rarely, its total obliteration. 3d. The more or less complete obliviousness of the thoughts and events of the mesmeric state, while awake, although the memory of the events of the natural state is strong in the artificial state. 4. The retention of locomotion and the facility of being led into suggested dreams, are also curious effects of the mesmeric action. Nothing is too high for the daring, or too absurd for the belief of the dreamer. But all the mesmerized patients are not susceptible of this influence. A few subjects resist, even when asleep, all attempts to mislead them, although they present most of the other peculiarities of somnambulism.

10. To this property of artificial dreaming may be referred the alleged miracles of *clairvoyance*, intuition, and prevision. The subject dreams that he sees, and the questioner is deceived, by his confidence, his plausibility, and his ordinary character. He knows him to be honest, and he does not perceive that he is himself led astray by his uncorrected imagination. There is all the effect of a fraud, without intention to mislead, and without blame.

11. The mesmeric effect is usually producible within ten minutes, and at the first sitting, but some persons have yielded only after long and repeated trials. In general, unless very marked effects are exhibited within half an hour, all subsequent attempts to mesmerize are fruitless.

12. The mesmeric sleep may be dissolved by time alone, the natural duration of the paroxysm lasting from thirty minutes to nearly five hours. The fear of not escaping from the spell, in the event of death, or absence, or loss of power of the magnetizer, is therefore not well founded.

13. The artificial solution of the mesmeric sleep requires sometimes only a single wave of the hand, sometimes many. The mean time is about two minutes.

14. Independently of the voluntary aid of the mesmerized subject, the time taken to dissolve the sleep is very sensibly affected by the distance from him. Thus, in contact, a case consumed 4′ 4″; at two yards, 7′ 30″; at four yards, 16′ 45″.

15. Sex does not appear to exercise any very marked influence on the mesmeric susceptibility.

16. Age is a more modifying cause than sex. Though no age is exempted, the very young and old seem least susceptible; and the period of life between twelve and twenty is that most favourable to the mesmeric influence.

17. Of the temperaments, the nervo-sanguineous seems most liable to the mesmeric action.

18. Although without an exception, so far as I can discover, mesmerists agree in believing that a sound state of health is unfavourable to the success of their operations, I have found it most conducive to well marked mesmeric results. Of twenty-six somnambulists, nineteen were in good, and seven in bad health.

19. The mesmerizing power seems to be very generally possessed, but the susceptibility to soporose mesmeric impression is confined to a few individuals, being about one in seven or eight of those subjected to the trial.

20. The *rapport*, *relation*, or *communication*, supposed to have an absolute existence, dependent on the mesmeric fluid, seems to be entirely voluntary on the part of the patient, and to rest on his knowledge of its supposed necessity. It is, therefore, a delusion; but one of the greatest convenience to the public exhibitors of mesmeric wonders.

21. The delusion as to the "*rapport*" is one of the many hallucinations of the mesmeric state, for which the subject of it is no more answerable than for any of the wild and monstrous dreams to which the disordered fancy may be led, in that unnatural condition both of mind and body. This truth is clearly proved by analogical cases of insanity, where similar delusions continue for years.

22. The mesmeric state curiously modifies the condition of the senses. Sight, hearing, and touch are usually improved, taste, smell, and sense of pain as commonly impaired.

23. As the sense of touch and of pain are so diversely affected by mesmerism, we are led to regard them as independent senses; probably, therefore, supplied by separate nervous fibres. Such an inference ought to have been made before, for many organs have the sense of pain, but not the sense of touch. The presence of a poison will give pain to the stomach or intestines, which do not perceive the motions of the worms that infest them. If this view be correct, the sense of pain is a sixth sense.

24. *Many* of lhe feats of the *clairvoyants* are the result of the sharpened hearing, which enables them to detect objects by the sounds they make. They really believe they see them, and so does the exhibitor, although he aids them by handling audibly the various objects. Thus he opens and shuts a pencil, a penknife, or a spectacle case, and rubs a stick, or a sheet of pasteboard. He always makes as much noise as possible with every thing, and he generally asks the producer of a marked card to explain the words or device to him.

25. As we cannot believe in mesmeric "rapport," so we are not able to credit the existence of any peculiar sympathy between the operator and subject. Untrained or ignorant patients never show sympathetic phenomena. I have been pinched, and hurt otherwise, a great many times, without observing any suffering on the part of my subjects, until they were taught to believe that such a relation existed; and then they very honestly felt hurt, as people do in dreams—a kind of imaginary suffering.

26. The phrenological phenomena of mesmerism, when rigidly examined, are found to consist, as do most of the mesmeric wonders, of "such stuff as dreams are made of." The *excitement* of the brain *is general*, the *direction* of that excitement is *given* by *the mesmerised person's knowledge of phre-*

*nology;* but the patient is not in any case aware of his mental co-operation. This singular delusion or misapprehension, runs through nearly the entire subject of mesmerism; most of the phenomena of which are a strange mixture of physical impulse and mental hallucination. Phrenologists alone feel the phreno-mesmeric excitement. Persons partially acquainted with phrenology, experience it only as to the organs known to them; while those who are totally ignorant of the subject, present no local manifestations, until they are taught, either awake or asleep, what they should know, and what they should do. The displacement of old organs, in one city, their retention of location in another, and the adherence of the patients to the peculiar and dissimilar systems of phrenology, which they have, respectively, been taught, show clearly, that the direction of the cerebral excitement is personal and arbitrary; while the new maps of the cranium, so widely different from each other, leave us no longer in the least doubt as to the delusive source of the compound science of phreno-mesmerism.

27. The mesmeric influence is the effect of what the natural philosophers call *induction.* The will of the operator acts solely on himself; his altered system reacts on the subject of the experiment, by an unexplained power, analogous to the equally inexplicable *induction* of the mechanicians, and the *presence* of the chemists.

28. Mesmerism may be sometimes usefully employed to allay nervous irritation, procure sleep, and obtund nervous sensibility, during surgical operations; but from the fewness of susceptible persons, it can be used very seldom for such purposes. In all other cases it appears to be of little use; and so far as I know, has never cured any serious disease. On the other hand, it sometimes, especially in unpractised hands, produces frightful disorders both of mind and body, and should therefore be resorted to solely for proper and important purposes, and then only with due precaution.

29. The cases of natural somnambulism, so like those of the mesmeric state, the permanent magnetic power of some individuals, the relief afforded to paralysis and stupor, and the restoration from *natural* somnambulism by mesmeric passes, go far to show that the disturbance of the nervous system, which is produced by mesmerism, may and does occur in certain stages of disease, and is not unfrequently present in nervous affections where we have not hitherto suspected its coincidence.

30. Mesmerism may, for the above reasons, be employed to relieve, temporarily, affections of a nervous character, when the usual means fail; but it should be used always with caution, and only when the failure of all ordinary measures renders its application a matter of necessity.

31. The claims to a peculiar *medical intuition,* set up by magnetized persons, or their exhibitors, are destitute of foundation. The pathology is usually absurd, the prescriptions are inefficient, dangerous, or ridiculous, and, after sixty-eight years, mesmerism has not detected a new theory of disease, or suggested one useful remedy.

In conclusion—I may be, perhaps justly, charged with giving to the subject of mesmerism an undue importance, and bestowing on it a disproportionate share of time and attention. The results, being chiefly negative, add almost nothing to our stock of knowledge, and the pretensions now *demonstratively* overthrown, being discarded already by common sense, and the antecedent labours of others, scarcely deserve, in the opinion of the world, a passing notice. But I think I am justified in my laborious investigation, by the interest still felt in the subject, over a large part of the civilized world, by the want of digested and comprehensive facts, and by the bearing of the phenomena on the practice of medicine, and on the physiology of the nervous system. Perhaps, too, it may not be unimportant to the guardians of public and private morals, the administrators of justice, and the conservators of family and educational discipline, to learn what unsuspected physical agents are at work on the human frame, at all times, and in all places. They may thus be enabled, not only to guard against abuses, but to make indulgent and charitable estimates of the character and extent of crime and error.

Doubtless, the mesmerists will say that I pay too little attention to the testimony of others on many of the points in which I differ from them, and others may allege that for all that part of the subject which I admit to be true, I give too much weight to my unsustained labours and observations. To both, I may with truth, and without undue pretension reply, that I did not expect to settle any question *definitively* by these researches. They were made carefully and honestly, and the results set down without exaggeration or extenuation, for the purpose of making as close an approximation to an obscure truth, as the time and opportunity would permit. Others, following in the same exact path, may enforce or weaken my conclusions ; but sure I am, that it is only thus that we shall finally settle these vexed questions, and not by opinions founded on unrecorded observations, or vague generalities derived from loosely kept records. While I find volumes of conclusions, I discover no tables to which I can refer for support or refutation. I see many edifices, but I discover no foundations for them, and naturally infer that, as they rest on no solid bases, they are without weight, and made of imagination.

As to the charge of refusing the testimony of others, I answer that their evidence is so conflicting as to destroy itself. The most substantial proof, that of distinguished medical men, is usually on my side, and if I have not availed myself of that, how can those complain who give *opinion* on the other side? Few are competent to observe, in a question involving medical knowledge and scientific attainment. He who would truly understand such phenomena, must know all that is known of the nervous system, and much that is taught as physical science. He must have studied, also, the human mind in health and disease, and have examined the kindred complaints of somnambulism and catalepsy. Now, it is not a little remarkable that the authors who have written in favour of the higher claims of mesmerism, have not been thus prepared, while the more accomplished observers have decided against those claims. Let me illustrate this farther. Phenomena are observed in the heavens—among the stars. Every one sees them—but to whom do we look for the explanation by which these phenomena are fashioned into facts. For how many thousand years did the constellations glide across the zenith, in nightly brilliancy, observed by millions of eyes, before the splendid phenomena assumed to the human understanding the shape of a fact. Until explained by Copernicus, it was a bright illusion—*the very opposite of that which it seemed.* If this illustration does not lessen the confidence of ignorant observers in their powers of discrimination, I am at a loss for means to teach them humility, which can alone give much value to the observations of any one, however otherwise prepared for investigation. That sleepers often describe well distant places and events, is true ; but does it follow that they obtain the knowledge by spiritual inspection ? Or are they indebted to other and more intelligible means of discrimination ? It is not less true, that there is sometimes the manifestation of strong personal sympathy between mesmerizer and subject, but are there not unexamined sources of error in the most obvious explanation of this phenomenon? The dispute is less as to the appearances, than as to the view to be justly taken of them. The vast and airy beings that darkened for ages the skies of the Brocken Mountain, were the wonder and terror of the ignorant peasantry, until more competent observers proved them to be the shadows of human beings, cast by the rising and setting sun in exaggerated volume on a screen of clouds. That which had been a frightful phenomenon, became an agreeable fact. The shadowy things of artificial somnambulism have long enough displayed their visionary forms on the sky of human wonder. It is time to give them that true import which will take them from the mountebank and pretender, and place them in the hands of philosophy. If I can believe that I have done so much as to bring philosophy to the task, free from prejudice and restraint, I shall be satisfied that my labour has not been in vain.

